# Recent developments in cancer diagnosis and treatment using nanotechnology

**DOI:** 10.1097/MS9.0000000000002271

**Published:** 2024-06-17

**Authors:** Olfa Ouled Ltaief, Ilham Ben Amor, Hadia Hemmami, Wiem Hamza, Soumeia Zeghoud, Asma Ben Amor, Mourad Benzina, Ali Alnazza Alhamad

**Affiliations:** a Water, Energy and Environment Laboratory, National School of Engineers of Sfax, University of Safx, Safx, Tunisia; b Department of Process Engineering and Petrochemical, Faculty of Technology; c Renewable Energy Development unit in Arid Zones (UDERZA), University of El Oued, El Oued, Algeria; d Department of Chemistry, Faculty of Science, University of Aleppo, Aleppo, Syria; e Department of Technology of organic synthesis, Ural Federal University, Yekaterinburg, Russia

**Keywords:** nanomaterial synthesis, targeted drug delivery, nanotechnology in cancer, therapeutics

## Abstract

The article provides an insightful overview of the pivotal role of nanotechnology in revolutionizing cancer diagnosis and treatment. It discusses the critical importance of nanoparticles in enhancing the accuracy of cancer detection through improved imaging contrast agents and the synthesis of various nanomaterials designed for oncology applications. The review broadly classifies nanoparticles used in therapeutics, including metallic, magnetic, polymeric, and many other types, with an emphasis on their functions in drug delivery systems for targeted cancer therapy. It details targeting mechanisms, including passive and intentional targeting, to maximize treatment efficacy while minimizing side effects. Furthermore, the article addresses the clinical applications of nanomaterials in cancer treatment, highlights prospects, and addresses the challenges of integrating nanotechnology into cancer treatment.

## Introduction

Highlights

- Nanoparticles for cancer diagnosis
- Synthesis of nanomaterials.
- Nanoparticles in imaging enhancement.
- Drug delivery via nanotechnology.
- Mechanisms of targeting cancer cells.
- Real-world clinical applications.

Despite notable progress in the field of medical research and Technology, cancer continues to be an illness that has a restricted range of therapeutic options. The occurrence of metastasis and cancer recurrence significantly contribute to both disability and mortality. However, the precise mechanisms behind these processes have yet to be fully understood^1,2^.

Existing methods for diagnosing cancer involve the use of imaging techniques, laboratory testing, and the examination of tissues and cells for their physical characteristics. This approach is generally regarded as very dependable for most cancer diagnoses^3^. Pathological features, including immunohistochemistry (IHC) analysis, histological changes, and mutational and molecular genetics studies, are also utilized in the diagnosis of cancer^4^. Standard cancer treatment typically involves surgical excision, chemotherapy, radiation therapy, and biological therapy. Surgery is a highly efficient method for eliminating malignant solid tumors, particularly when performed during the initial stages of cancer progression. Combined therapy encompasses a range of treatments, including surgical intervention, chemotherapy, and radiotherapy. Chemotherapy has gained popularity over the years due to its ease and convenience in treating patients with cancer^5^.

Chemotherapy and radiation treatment alone can only cure 5% of cancer patients; however, surgery can cure around 45% of cancer patients^3^. The remaining patients do not get well from this illness. Of all the cancer therapies available, although chemotherapy is a commonly used cancer treatment, it nevertheless has some serious drawbacks. The inability to diagnose cancer early, the dispersion of non-targeted drugs, etc., are the limits of cancer therapy^6^. Since the non-targeted medication concentration spreads throughout the body, excluding the tumor site, it is less able to monitor the drug’s effects on the body and treat the tumor site. One of the most concerning aspects of employing conventional cancer therapy is that the drug’s toxicity level impacts both healthy and tumor cells. Heart failure is one of the major consequences that the poisoning may induce. The scientific community is working to develop novel therapies employing cutting-edge nanotechnology to address these issues. Dendrimers, polymeric, magnetic, liposomal, and nucleic acid-based nanoparticles are among the most widely employed types of nanocarriers^7,8^. By reducing the drug’s toxicity level and, therefore, raising its effectiveness and enabling it to reach a steady state therapeutic level over a longer period, nanocarriers may enhance the therapeutic index of presently existing medications. The area of nanotechnology is always evolving and is quickly emerging as the most vital component in the fight against cancer. This review article aims to elucidate the pivotal role of nanotechnology in advancing cancer diagnosis and treatment, explore the functional diversity of nanoparticles in oncologic applications, and assess future directions and challenges in nanomedicine for cancer care.

## Importance of nanoparticles in the diagnosis of cancer

Nanoparticles, including magnetic, metallic, metal oxide, polymeric, graphene, quantum dots, liposomes, fullerene, dendrimers, and carbon nanotubes, are used in the diagnosis of breast, colon, and cervical cancer….. etc^9,10^. They also play a crucial role in imaging capabilities. Nanoparticles persist in the bloodstream for extended periods before reaching their intended cellular targets, navigating biological barriers like cell wall membranes. Cancer-specific antigens can be connected to nanoparticles to enhance the affinity and identification of cancer cells. Current research shows the potential of sensors and nanoparticles to improve tumor detection sensitivity and cancer diagnosis^11^. Metal nanoparticles are more sensitive in identifying cancerous cells due to their ability to be linked with cancer-specific antibodies. Nanocomposites incorporating multiple types of nanoparticles, including cobalt, silver, platinum, copper, and gold, exhibit superior potential for scanning and detecting cancer. These nanomaterials have distinct chemical, physical, and magnetic characteristics, enabling resolving imaging scanners with enhanced contrast, increased sensitivity, and regulated distribution within living organisms. These characteristics can result in clinical benefits such as early identification, immediate disease evaluation, and customized medical treatment^12^.

## Nanomaterials synthesis

Nanomaterials can be synthesized using either conventional methods or environmentally friendly technology. The synthetic techniques encompass thermal decomposition, chemical vapor deposition, solvothermal preparation, hydrothermal treatment preparation, combustion, pulsed ablation using lasers, templating, microwave synthesis, traditional sol-gel synthesis, and gas phase method. Every technique possesses its own merits and drawbacks^13^.

## Function of nanoparticles in contrasting agents and image enhancement in cancer

Research primarily focuses on improving cancer imaging techniques, with nanoparticles playing a crucial role in diagnosing and treating cancer, particularly as photosensitizers, and their value in personalized therapies based on nanotechnology in medicine^14^. MRI is a crucial application that utilizes magnetic nanoparticles. Iron oxide nanoparticles (IONPs), are widely used in liver and lymph node imaging and cell tracking^14^. Nanotheranostics, a promising strategy for cancer treatment and imaging, utilizes intricate nanoparticles made of polymeric substances, bismuth, proteins, gold, and calcium phosphate. These nanoparticles have been shown to improve screening, imaging, and early identification of patients with colorectal cancer (CRC). Colonoscopy is the primary method for diagnosing CRC, but alternative techniques like transrectal ultrasonography, MRI, and computed tomographic colonography (CTC) have also been employed for CRC detection^15^. MRI has made significant progress in diagnosing CRC, but it’s not highly efficient for detecting small polyps. Alternative methods like Fluorodeoxyglucose-positron emission tomography and (FDG-PET) have been used, but they have shown inaccurate results due to radiation exposure^15^. Metallic nanoparticles and cancer-specific antibodies have improved the detection of breast and colon cancer in urine or blood tests. Proteins and biomarkers present on cell membranes or extracellular environments enable precise diagnosis across imaging modalities like computed tomography (CT) and MRI. Photoacoustic imaging offers high resolution and strong contrast in optics, while Raman imaging is an efficient method for identifying cancer cells. These techniques enhance precision in tumor excision and can be validated through histological examination^15^.

Chemotherapy, despite its high toxicity and detrimental adverse effects, is a primary treatment approach employed for treating different forms of cancer. Multiple investigations have been undertaken to identify precise transporters for this treatment^16^. Magnetic microcapsules and nanoparticles have shown improved therapeutic effects in chemotherapy for liver and brain cancers. Prodrugs can enhance drug distribution, biodegradable polymers, and macromolecular matrix approaches. Magnetic nanoparticles have minimal toxicity and have numerous biomedical uses, especially in cancer diagnosis and treatment^16^.

Currently, extracellular vehicles (EVs) are widely recognized as nanocarriers that can help to some extent in resolving the problems related to chemotherapy. According to reports, EVs can effectively traverse the natural membrane due to their smaller dimensions and have the opportunity to stay in the blood circulation for a long time, allowing them to target cells more effectively. EVs exhibit reduced toxicity and immunogenicity compared to alternative medication delivery strategies. Research has demonstrated that drug delivery via electric vehicles is successful in treating cancer, as evidenced by positive outcomes in both laboratory and live tumor and cancer models. EVs offer numerous benefits, including enhanced loading and target efficacy, improved circulation duration, and reduced occurrence of side effects. The utilization of diverse nanoparticles in the diagnosis of cancer is documented in Table 1.

**Table 1:** Utilizing nanoparticles to diagnose cancer^17^

Type of nanoparticles	Properties	Cancer diagnosis example
Lipid-based nanoparticles	Superior biocompatibility and little toxicity as compared to inorganic nanoparticles	The process of attaching anti-HER2 antibodies to phospholipid-coated quantum dots (QDs) demonstrated the capability to specifically target tumors that are positive for the HER2 protein
Carbon-based nanoparticles	The physio-chemical features of this substance are exceptional, characterized by its ability to penetrate the cell membrane easily, its large surface area, and its excellent ability to load drugs	Both in-vivo and in-vitro investigations demonstrate that nanodroplets serve as a very efficient contrast agent for both photoacoustic and ultrasonic imaging
Polymeric nanoparticles	Encased inside a polymer shell	Targeted tumor delivery is achieved by the use of block copolymer-coated nanoparticles conjugated using pigment molecules and RGD peptides
Ceramic nanoparticles	Excellent biocompatibility	Gold nanoparticle-based photothermal treatment is being considered for clinical research to effectively destroy recurrent head and neck cancers and for cancer imaging purposes
Metallic nanoparticles	Magnetic nanoparticles have a vital role in the identification and prevention of metastatic breast cancer	Utilizing gold nanoparticles for Raman imaging

RGD peptides are short amino acid sequences that contain the amino acids arginine (R), glycine (G), and aspartic acid (D); The name “RGD” comes from the initials of these three amino acids.

## Frequently used nanoparticles in the therapeutic of cancer

Nanotherapeutics is the term used to describe the use of nanoparticles in the field of medical diagnosis^18^. In recent times, nanotechnology has advanced to enhance clinical therapeutics, owing to its heightened sensitivity and capacity for early identification of cancer. Various types of nanomaterials, including quantum dots, polymeric nanoparticles, dendrimers, and carbon nanotubes, are utilized in cancer therapeutics. To improve the ability of nanoparticles to detect cancer, they can be combined with antibodies, aptamers, peptides, carbohydrates, and other additional little compounds that selectively target molecules and enable them to reach the desired location^19^. Research has been carried out employing gold nanoparticles for cancer diagnosis. Au-doped nanoparticles exhibited remarkably high luminous intensity^20^. In addition, fluorescence has been employed to detect the cancer biomarker carcinoembryonic antigen (CEA) using color visualization. An identifiable red color change was used to establish the limit of detection (LOD), which was found to be 10.00 ng/ml, while an LOD of 0.10 ng/ml was attained by differentiating fluorescence intensity. An assessment was conducted on the feasibility of this approach using actual clinical specimens^21^. According to reports, using an immunoassay technique provides better control over the placement of antibodies and has the potential to accurately analyze the liver cancer biomarker Hsp90α^22^. In addition, miR-377-3p and miR-381-3p were employed as diagnosis biomarkers for CRC^22^.

Because of their quick detection times, nanoparticles made of gold, silica, silver, iron, and magnetic oxide are used in the identification of cancer and economic nature. Furthermore, When compared to radiotherapy and chemical-based treatments, they have fewer side effects. Electrochemical biosensors are characterized by their simplicity, affordability, and effectiveness, making them a highly valuable approach to cancer therapeutics^23^. Functionalization of nanoparticles can enhance their ability to detect cancer. As an illustration, in the identification of breast adenocarcinoma cells, cancer-specific antibodies were linked with polyethylene glycol (PEG). The antibody-PEG complex was affixed to the surface of the nanoparticles by a group that contains sulfur located at the far end of the PEG linker^19^.

### Metallic nanoparticles

Metallic nanoparticles, ranging from 1 to 100 nm in size, are classified into four categories: metallic nanowires, metallic nanoplatelets, metallic nanoparticles, and metallic nanostructures. Due to their intense energy, they clump together, promoting coalescence through thermodynamic processes. To stabilize metallic nanoparticles in liquids, two methods are commonly employed: static stabilization and steric stabilization. Static stabilization involves attaching negatively charged ions to the surface of the nanoparticles, forming an electrical double-layer. This electrostatic repulsion between particles helps prevent their aggregation. On the other hand, steric stabilization entails coating the nanoparticles with a surfactant, polymer, or ligand. This protective layer creates a physical barrier that inhibits the close approach of nanoparticles, thus preventing their agglomeration^24^.

#### Platinum nanoparticles (PtNPs)

PtNPs have significant medical applications due to their antioxidant properties, which suppress tumor growth. They also enhance tumor targeting through the use of ligands that bind to functionalized metal PtNPs. PtNPs also promote superior drug release and delivery effectiveness. However, current research shows harmful consequences due to the nanoparticles’ size, which tend to concentrate in vital organs and cells^25^. Table 2 exhibits the utilization of platinum-based nanoparticles in cancer therapies.

**Table 2:** Use of platinum-based nanoparticles in many cancer therapy methods

Cancer	Nanoparticle type	Application	Results	Ref.
Colon cancer cells	Pt/MgO nanoparticles	Induced apoptosis in colon cancer cells	Colon cancer cells have a decrease in the expression of Bcl_2_. Elevated expression of Bax and p53 in colon cancer cells under laboratory conditions	^26^
Colon cancer cells	Gold-decorated palladium and platinum nanoparticles	Enhanced the efficacy of simulated proton therapy for anti-cancer treatment	Apoptosis-induced cell death in cancer cells (*in vitro*)	^27^
Glioblastoma and melanoma cells	Ag-Pt nanoparticles	Managing the cancer cell types glioblastoma and melanoma	There were no harmful effects seen in healthy cells when tested in a laboratory setting *(in vitro*)	^28^
Neuroblastoma cancer	PtNPs	Stimulation of cancer cell apoptosis	The PtNPs caused cancer cells to undergo apoptosis, resulting in their death. Additionally, they generated oxidative DNA damage *in vitro*	^29^

Ag, silver; Bax, is a pro-apoptotic protein and a member of the Bcl2 family; Bcl_2_, B-cell lymphoma 2 proteins; MgO, Magnesium oxide; p53, is a tumor suppressor protein that acts as a central regulator of cellular responses to various stress signals; Pt, platinum; PtNPs, platinum nanoparticles.

#### Gold nanoparticles

Because of their numerous distinctive qualities, gold nanoparticles (AuNPs) are useful for a range of medical purposes. AuNPs are diminutive molecules that exhibit numerous distinctive features suitable for imaging techniques. Gold nanoparticles in cancer imaging techniques provide extended circulation durations in the bloodstream, resulting in improved tumor targeting and higher-quality diagnosis. In addition, AuNPs have a broad range of uses, including nucleic acid delivery, medication administration, radiotherapy, and photothermal ablation^26^. AuNPs can be produced in diverse dimensions and configurations, exhibiting exceptional adaptability^26^. Furthermore, AuNPs have exhibited low cytotoxicity and enhanced biocompatibility, rendering them very attractive contenders for clinical applications (Table 3).

**Table 3:** Use of gold nanoparticles in tumor imaging and identification

Nanoparticles	Nanoparticle type	Imaging	Application	Ref.
Gold nanoparticles	Spheres	X-ray imaging	High payload delivery	^30^
	Rods, shells, labeled spheres	Fluorescence imaging		
	Stars, spheres	Imaging using surface-enhanced Raman spectroscopy	Produces a powerful electromagnetic signal	
	Clusters, spheres, rods, shells	Photoacoustic imaging	Demands high levels of absorption within the near-infrared (NIR) range	
	Primarily spheres	Optical imaging	The light utilized falls within the near-infrared (NIR) spectrum	

The Food and Drug Administration (FDA) has approved many therapeutic items based on AuNPs, which are currently accessible in the market. Additionally, several other formulations of AuNPs are currently undergoing experimental phases. Table 4 shows how gold nanoparticles are used in various cancer identification and diagnosis methods.

**Table 4:** Effects of gold nanoparticles on various cell types

Type of cell	Application	Results	Ref.
Finding a biomarker for cancer carcinoembryonic antigen (CEA)	Fluorescence of resonance energy transfer (FITC) via Fluorescein isothiocyanate (FRET)	More selectively, fluorescence FITC employing the FRET approach may identify cancer cells (*in vitro*, *in vivo*)	^20^
MicroRNA biology	Detection of miRNA-155	Under ideal experimental circumstances, it is capable of detecting cancer (*in vitro*, *in vivo*)	^10^
Oral squamous cell carcinoma	Gold nanorod assay in conjunction with nano-ELISA	The sensitivity of cancer analyses was increased using enzyme-linked immunosorbent assay (ELISA) (*in vitro*, *in vivo*)	^27^
Prostate-specific antigen	Nanomaterial based on immunosensor	Superior specificity, sensibility, and durable-term stability immunosensor for in-vitro cancer bioassay analysis	^28^

### Magnetic nanoparticles

Magnetic nanoparticles are characterized by their small dimensions (10–50 nm), strong affinity for binding, and possession of various advantageous attributes, including the existence of holes, protons, electrons, and both positive and negative charges. Moreover, they are harmless and compatible with living organisms. They are commonly employed as a labeling bioconjugate for the detection of cancer biomarkers (Table 5). Furthermore, nanoparticles have a low detection limit and shorter test duration due to their interaction in a liquid solution rather than on an electrode’s surface. They have significant saturation magnetization, allowing particles to pass through tissues and organs easily. They exhibit stability even in aqueous solutions with a neutral pH of 7. Nanoparticles can be administered intravenously for cancer identification and can influence cells and transport medications to specific areas. They are ideal for MRI and can be easily manufactured and customized to meet individual needs. They are also safe and free from harmful substances, making them suitable for use in medical and environmental contexts. They can be easily manufactured and customized to meet individual requirements, making them an excellent choice for various applications.

**Table 5:** Applying magnetic nanoparticles for cancer screening and detection

Type of nanoparticle	Cancer cells	Applications	Ref.
Superparamagnetic iron oxide nanoparticles	Pancreatic cancer cells	MRI-based pancreatic cancer diagnosis and the possibility of early detection with focused techniques	^31^
Surface-modified magnetic nanoparticles	Colon cancer cells	For in-vitro colon cancer cell therapeutics	^31^
Magnetic nanoparticles	Liver cancer cells	Improved in-vitro identification of liver malignant cells	^21^
Magnetic nanoparticles	Brain cancer cells	*In vivo*, cancer identification and therapy using magnetic nanoparticles as contrast materials	^32^
Magnetic gold nanoparticles	Breast cancer checks	Breast cancer detection with ELISA, particularly for those with HER2 breast cancer	^33^

ELISA, enzyme-linked immunosorbent assay.

Nanoparticle synthesis involves various techniques, allowing for precise characteristics like enhanced magnetic potency or enhanced drug transportation. Control over dimensions, configuration, and constituents allows for selective reactions with chemicals or cells. Functionalized magnetic nanoparticles, using magnetic fields or near-infrared light, have been used to treat cancer by enhancing their ability to interact with specific chemicals or cells^29^.

Ferrous oxide nanoparticles are beneficial for in-vivo research due to their high degradability and cytotoxic action. They are commonly used in cancer treatment using magnetic fields to produce oxygen radicals and can be manipulated from a distance using an external electromagnetic field. These nanoparticles can be locally hazardous due to reactive nitrogen and oxygen species but are beneficial for treating tumors. Iron oxide nanoparticles, loaded with anti-cancer medications, have advantages over traditional treatments due to their ability to be regulated from a distance, as demonstrated in a breast cancer mouse model^30^. During the progression of breast cancer, there is an observed overexpression of miRNA-155, which is recognized as a biomarker for this type of cancer. Mapping miRNA-155 is challenging because of the absence of highly sensitive approaches. A novel methodology has been devised to address this issue, enabling the fast production of magnetic nanoprobes capable of quantifying miRNA-155^34^.

### Polymeric-based nanoparticles (PNPs)

PNPs are solid substances with dimensions ranging from 1 to 1000 nm, exhibiting strong mechanical, electrical, thermal, and optical properties. They can be combined with drug delivery methods, fluorophores, and active pharmaceutical ingredients for improved efficacy^35–37^. Nanoparticles are used in drug delivery to protect medication molecules. Biopolymers, including PNPs, are used for pharmaceutical administration, including chemotherapeutic medicines, antiviral compounds, essential nutrients, antioxidants, and plasmid DNA. PNPs are produced from biodegradable and non-biodegradable synthetic polymers. Non-biodegradable polymers like poly-acrylates are used for drug administration through the skin, while biodegradable polymers like poly(lactic-co-glycolic acid (PLGA) are used for transdermal drug delivery^35^. PNPs have been employed to enhance optical and magnetic resonance imaging techniques to diagnose brain cancer^38^. Dendrimers are a type of three-dimensional core-shell polymer that possesses the capacity to cross the blood-brain barrier, hence enhancing their targeting capabilities. Dendrimers, namely poly-amidoamine, are extensively utilized for drug delivery purposes. In particular, dendrimers that are linked with tamoxifen serve as effective drug carriers^38^. A surfactant is frequently used during the manufacturing of polymeric-based nanoparticles to help reduce the solution’s surface tension. Subsequently, the surfactant is combined with the monomer, which serves as the fundamental unit of the polymer. Subsequently, the monomer undergoes polymerization, resulting in the formation of nanoparticles.

Polymeric nanoparticles exhibit certain drawbacks, including limited stability and an aggregation propensity. Their qualities might provide challenges in certain applications, such as drug delivery^39^. In addition, the synthesis of nanoparticles based on polymers might be challenging due to the requirement for precise control of reaction conditions. Polymeric nanoparticles are susceptible to oxidation, which can restrict their application in certain contexts. Polymeric-based nanoparticles pose challenges for scaling up in industrial applications due to the need for meticulous monitoring of reaction conditions. Protease-activated near-infrared fluorescent polymeric nanoparticles, doxorubicin-loaded, have been utilized for cancer detection and treatment^39^.

### Metal oxide nanoparticles

Metal oxide nanoparticles are particles of metal oxides that are extremely small in size. Metal oxide nanoparticles, such as nickel oxide (NiO), zinc oxide (ZnO), manganese dioxide (MnO_2_), iron(III) oxide(Fe_2_O_3_), titanium dioxide (TiO_2_), and cobalt oxide (Co_3_O_4_), are composite materials composed of many metal oxides^13,40^. These nanoparticles have been recently employed in electro-analysis to identify and measure biomolecules. Utilizing metal oxides for biomolecule detection provides benefits like enhanced biocompatibility for enzymes, hence enhancing detection precision. Metal oxide nanoparticles can modify their structure, hence influencing the electrical conductivity and chemical properties of nanoparticles^41^. Moreover, oxides of transition metals can decompose diverse colors when exposed to sunlight or UV light. The use of ecologically friendly and sustainable synthesized biological components, such as metal oxide processes, is becoming more and more prominent. The green approach has numerous advantages, including the utilization of inexpensive constituents obtained from diverse plant sources, which may be readily expanded to produce Nanostructures of transitional metal oxides^42^. The nanoparticles of metal oxide are widely used and have a variety of applications in therapeutic and therapeutic contexts. For instance, zinc oxide (ZnO) nanoparticles were employed as a remedy for cardiovascular illness associated with diabetes. This was done by injecting streptozotocin into rats with diabetes. The findings demonstrated that administering a small dosage of ZnO nanoparticles effectively shielded heart cells from harm by decreasing serum concentrations of cholesterol^33^.

### Quantum dots (QDs)

QDs are minuscule crystals that possess the capability to convey electrons. When exposed to UV light, QDs can generate light of several colors with exceptionally high energy. These QDs have numerous applications, such as in the advancement of solar cells, fluorescent biological markers, and nanocomposites. High-resolution cellular imaging has led to advancements in cancer diagnosis. The fluorescence characteristics of quantum dots undergo alterations when exposed to various substances. Furthermore, QDs possess an inert region on their outer layer, which allows for the convenient attachment of particular antibodies. QDs have been utilized in many bio-applications for imaging purposes. QDs have been used in drug administration and cancer therapy, particularly in treating lung cancer. They effectively eradicate bacteria-induced infections and suppress lung cancer cells’ P-glycoprotein DNA and protein activity by stimulating miR-34b and miR-185 production. These miR-34b and miR-185 targets for lung cancer treatment. Combining QDs with Camellia sinensis leaf extract has been shown to impede the lung tumor cell cycle and reduce cancer cell viability^43^. Another research investigation found that the application of uncapped cadmium telluride quantum dots (520Q cytotoxic, 580Q cytotoxic, and 730Q non-cytotoxic) caused oxidative stress in lung malignancies and human bronchial epithelial cells. Nevertheless, it was shown that 730Q had a discernible impact only when the exposure time was extended but not when it was shortened^44^. In addition to their numerous advantages, QDs include heavy metals like cadmium, which is a recognized hazardous substance and carcinogen. This presents potential risks for their use in therapeutic applications.

### Graphene

Graphene, commonly known as individual graphite atomic layers, has a planar structure composed of uniform hexagonal rings. It possesses exceptional thinness, transparency, and lightness and exhibits excellent thermal and electrical conductivity, rendering it a highly desirable substance for the identification and detection of cancer. Graphene possesses several advantageous characteristics, such as its ability to exhibit a bipolar transistor impact and generate significant quantum fluctuations^45^. These traits render it a promising contender for efficient cancer imaging. Graphene possesses a significant specific surface area, making it advantageous for the incorporation of anti-cancer medicines as a result of the existence of π-π stacking and hydrophobic interactions^45^. Graphene oxide, an oxidized version of graphene, has applications in cellular visualization, drug delivery, and cancer treatment. It improves the properties of complex materials, and a study showed it can identify cancer cells through isotope separation of graphene Au nanocrystals^46^.

### Fullerene

Fullerene is a carbon allotrope with a lattice-like structure used in chemical applications to encapsulate drug molecules for effective drug delivery. Its C60 fullerene has been used in oncology for cancer diagnosis and detection and in biosensors for detecting glucose concentrations in blood serum. Fullerene’s interconnected carbon atoms allow for efficient drug delivery and have been successfully used in biosensors^47^. He can be captive by subjecting C60 to heating in the presence of helium vapor and applying pressure, as demonstrated by another study^48^. A recent study has found that fullerenes and metal nanostructures possess reducing capabilities that enable them to defend people from internal and external threats generated by reactive oxygen molecules. Recent research has discovered that fullerenes and metallic nanomaterials can specifically eliminate unhealthy cells in tissues, hence preventing the development of prolonged inflammatory illnesses. Fullerenes and metallic nanomaterials possess significant prospects in the management of diseases accompanying aging^48^.

### Carbon nanotubes

Carbon nanotubes are cylindrical carbon atoms with exceptional thermal and electrical conductivity, resilience, and lightness. They are useful in rechargeable lithium batteries, electronic nanodevices, hydrogen storage, composite materials, biosensors, and touch screens due to their 1 nanometer diameter and significant length. Single-wall nanotubes have better properties than multi-wall nanotubes like silver or copper^49^. Furthermore, carbon nanotube biosensors have been employed for the detection of organophosphorous chemicals; as demonstrated in previous works, Carbon nanotubes and gold nanoparticles were placed onto a gold cable. An enzyme called acetylcholine-esterase was used in the biosensor. They were mounted on carboxylic-functionalized single-walled carbon nanotubes. These nanotubes were then attached to the cardiac electrode. Nafion was added to the electrode to prevent enzyme release and convert it into a sensor electrode. This nanosensor operates based on the mechanism of suppressing the acetylcholinesterase enzyme^49^. A research experiment was performed in which Carbon nanotubes with carboxyl functionality were introduced to a human T-cell cell line. This led to the activation of caspase-2 gene expression within the living cells. The findings demonstrated a marginal decline in the cellular survival of cancer cells when subjected to carboxyl-functionalized carbon nanotubes. The molecular study revealed an upregulation of Cas2 mRNA in the cancer cells that were subjected to treatment with Carbon nanotubes with carboxyl functionality^50^. Nanotubes of carbon have been utilized in the domain of cancer therapeutics and imaging due to their biological compatibility, thermodynamical characteristics, and flexible functionality^51^.

### Liposomes

**Figure 1:**
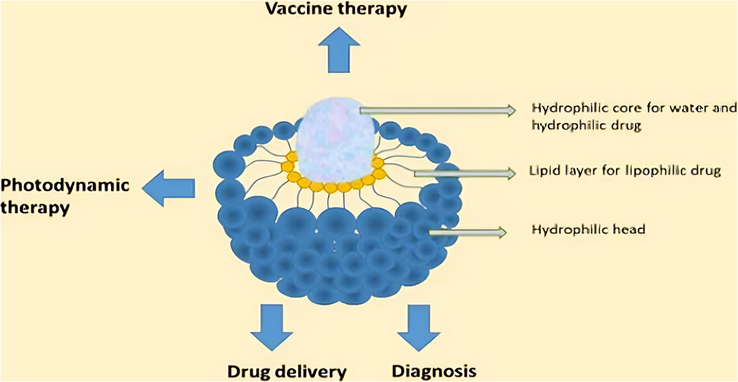
Applications and structure of liposomes (reproduced with permission from Noor *et al.* ^53^).

Liposomes are lipid-based nanoparticles consisting of a sealed lipid bilayer sphere containing an inner chamber for water solutions. The bilayer consists of two phospholipid sheets, a hydrophilic head area, and a hydrophobic tail, with hydrophobic tails exhibiting mutual attraction and membrane heads orienting towards water^52^. This structure consists of a bilayer of phospholipid molecules, which serves as An impermeable barrier that restricts the release of the internal solution to the exterior. Subsequently, the solution can be conveyed to the desired location by liposomes. They are innovative in the field of Pharmaceutical medication delivery methods and drug transfer mechanisms (Fig. 1). Liposome nanomolecules have shown a substantial influence on chemotherapy by enhancing selectivity, reducing cytotoxicity, and improving the solubility of hydrophobic medicines (Fig. 1). Microfluidics can be utilized to create small liposomes measuring around 50 nm in diameter, whereas giant liposomes measuring 75 nm in diameter demonstrated comparable drug retentivity in both *in vitro* and animal experiments. However, the extent of leakage was influenced by the size of the liposomes, with smaller liposomes exhibiting superior distribution throughout the tissues compared to larger liposomes^52^. Liposomes play a crucial role in chemotherapy administration, as they enhance transfection effectiveness and reduce chemo-resistant proteins. Studies have used liposomes to transport “Glucose regulated protein 78” and concentrate 1, 2-dioleoyloxy-3-trimethylammoniumpropane liposomes in cancer stem cells and mammary tumors, demonstrating effective transportation in both types of cancer^52^. Furthermore, magnetic liposomes were employed in the field of cancer therapy. Specifically, liposomal IR-780, an exceptionally stable nano-therapeutic drug, has been employed to cure brain tumors.

### Dendrimers

**Figure 2:**
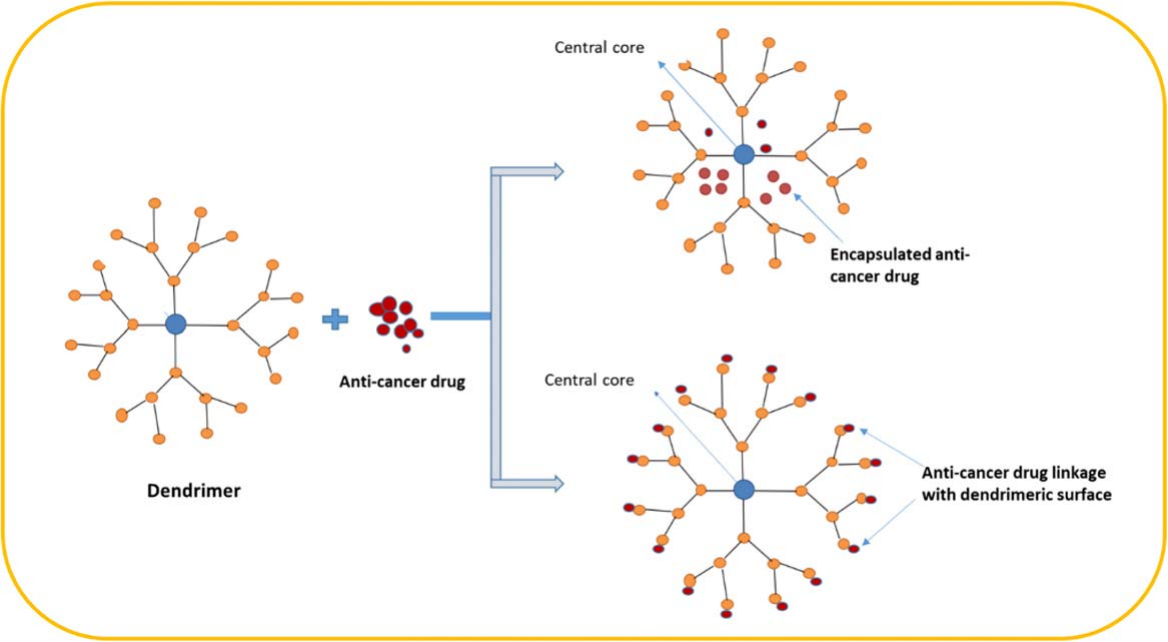
Dendrimers’ composition and use in drug conjugation and encapsulation (reproduced with permission from Noor *et al*.^53^).

Dendrimers are precisely engineered synthetic macromolecules characterized by an accurately specified three-dimensional arrangement and a large number of functional groups. Dendrimers, which are characterized by their compact size and dendritic architecture surrounding a core made of linear polymers, have found applications in the field of nanomedicine. Dendrimers serve as effective vehicles for the transportation of medications and genetics. Additionally, they may serve as antibacterial agents that are antibacterial and antitumor (Fig. 2). Dendrimers consist of three different layers: the center molecular, the middle layer, and the exterior layer. Furthermore, they possess viscosity, dissociation, and cellular properties. Dendrimers, due to their multipurpose structure, are valuable for creating advanced nanodevices in imaging and diagnosis. They have numerous applications in the medical field, including drug delivery, encapsulation, conjugation, nanocarriers, anti-cancer agents, gene delivery, photodynamic treatment, medical theranostics, and biosensors. Their contributions to materials sciences are substantial^54^. Furthermore, Dendrimers are used in the transportation of anti-cancer drugs due to their durability, solubility in water, and reduced immunogenicity. They can induce hypervascularization, increase tumor permeability, and impair lymphatic drain, facilitating passive targeting. Encapsulation or conjugation of dendrimers into therapeutic molecules may lead to safer and more effective medicines^45^. Dendrimers are created through divergent synthesis, a process where monomers are gradually incorporated into a central nucleus, forming a complex, branching architecture. Modifying these monomers allows for changes in the dendrimer’s dimensions, configuration, and chemical responsiveness. This intricate process requires careful management of reaction parameters. Dendrimers can be customized by changing monomer choice, reaction parameters, and monomer amount. They have unique properties, such as exceptional stability, durability, and solubility, making them useful for targeted drug delivery.

### Nanostructure lipid carriers (NLCs)

Nanocarriers based on lipids have demonstrated encouraging outcomes in the field of cancer therapeutics and treatment. Polymeric nanoparticles, formed by lipid-based chemicals or in conjunction with vectors like liposomes, ethosomes, and transfersomes, can potentially address challenges associated with drug absorption resistance in biological membranes. The synergistic impact of lipid-based nanocarriers is recognized for enhancing the effectiveness and precision of polymeric nanoparticles^55^. A study was performed to investigate the efficacy of a medication that combines nanostructured lipid carriers loaded with quercetin and coated with conatumumab, which is sensitive to reactive oxygen species and is being used to treat colon cancer^19^.

### Alternative nanoparticles and their utilization in diverse cancer cell types

The utilization of diverse nanoparticles has resulted in the proliferation of nanotechnologies across multiple domains, including pharmaceuticals, energy production, chemicals, drug administration, and enhancement of operational effectiveness. Various types of nanoparticles are currently employed for cancer cell detection and monotherapy. The functionalities of these nanoparticles can be determined by their size, kind, and structure^56^. Nanoparticles, including amino acids and semiconductor nanoparticles, are unique and deviate from ordinary particles. Nanorobots, miniature machines made of nanoscale molecules, can interface with the cellular barrier, providing direct access to cellular locations. They can enhance treatment effectiveness through advanced biological therapies and less invasive procedures. Currently, nanorobots are designed to identify 12 different types of cancer cells, demonstrating their potential in cancer treatment and screening. The effect of nanoparticles on different varieties of cancer cells, including cancer of the colon, neuroblastoma, breast cancer, glioblastoma and melanoma, oral squamous cell cancer, cervical cancer, prostate cancer, lung cancer, liver cancer, pancreatic cancer^57^, bladder cancer, brain cancer, cancer stem cells, and colorectal cancer^58^, has been studied. Table 6 provides a summary of the utilization of various nanoparticles in the field of cancer therapy.

**Table 6:** Using several kinds of nanoparticles in cancer treatment

Nanoparticle	Cancer type	Application	Ref.
Fumed silica nanoparticles	Detecting cancer pathways	For more accurate cancer detection, multi-site phosphorylated peptides may be effectively bound by nanoparticles (*in vitro*, *in vivo*)	^59^
miRNA	CRC	It may serve as a circulating biomarker for the early detection of CRC (*in vitro*).	^60^
Raman-active nanoprobe	Circulating cancer stem cells	Enhanced imaging via the use of the Raman imaging technique for the identification of cancerous cells (in laboratory settings and inside living organisms)	^61^

CRC, colorectal cancer.

### Creating nanomaterials to deliver drugs

Traditional administration methods like oral or intravenous injections have drawbacks, but controlled release systems offer numerous benefits, such as enhanced medicine efficacy, reduced side effects, and improved patient adherence to therapy, attracting medical professionals and patients, thereby creating significant market opportunities^62^. In recent years, various controlled release methods, such as micelles, nanoparticles, hydrogels, and electrospun barriers, were extensively studied to meet specific therapeutic application needs^63^. Poly (d,l-lactide-co-glycolide) mono/bicomponent electrospun polymer membranes have received approval from the FDA and have been employed as implant materials for an extensive duration. By changing the proportion of lactic acid and glycolic acid in the copolymer, these membranes’ mechanical characteristics and rate of decomposition can be customized^63^. To change the fibrous membrane’s swelling characteristics and regulate its rupture pattern, five distinct polymers, all of which have obtained FDA authorization for use in implantation, were included through the fiber layer as a secondary component. The polymers mentioned were poly(ethylene glycol) and poly(ethylene glycol)-b-poly(d,l-lactide)^63^. Hydrophilic polymer carriers often exhibit a rapid elimination profile, while hydrophobic polymers tend to liberate their loaded pharmaceuticals at a gradual rate. Extensive research has been conducted on multicomponent electrospun fibers that comprise both hydrophilic and hydrophobic polymers for the purpose of drug delivery^64^. In order to achieve prolonged therapeutic levels of anti-cancer compounds, a combination of rapid and gradual drug release mechanisms was employed using a hybrid material made of poly-caprolactone and gelatin. This material was utilized to encapsulate both free curcumin (CUR) and mesoporous silicon nanoparticles loaded with CUR^64^.

## Drug-targeted delivery system using nanotechnology in cancer therapy

Nanotechnology exhibits significant progress in the field of cancer therapy^23^. Nanotechnology enables the targeted delivery of therapeutic molecules to specific areas while minimizing harm to healthy cells^65^. Nanomedicine enhances the stability, solubility, medication half-life, and bioavailability of numerous chemotherapy medicines. Nanomedicine effectively decreased the maximum drug concentration, enhanced drug accumulation at specific locations, and raised the area under the curve^65^. The small size and distinctive nanoparticle coating of these particles enable the efficient transport of hydrophobic anti-cancer medications to specific areas in the body while minimizing their recognition and elimination by the immune system.

Nanoparticles (NPs) can enhance medication accumulation in cancer cells by increasing their Permeability and retention, a phenomenon referred to as improved retention and accessibility (EPR). In conclusion, The utilization of NPs in conjunction with anti-cancer drugs can augment the effectiveness of treatment by mitigating adverse effects via target-ligand interactions^66^.

**Figure 3:**
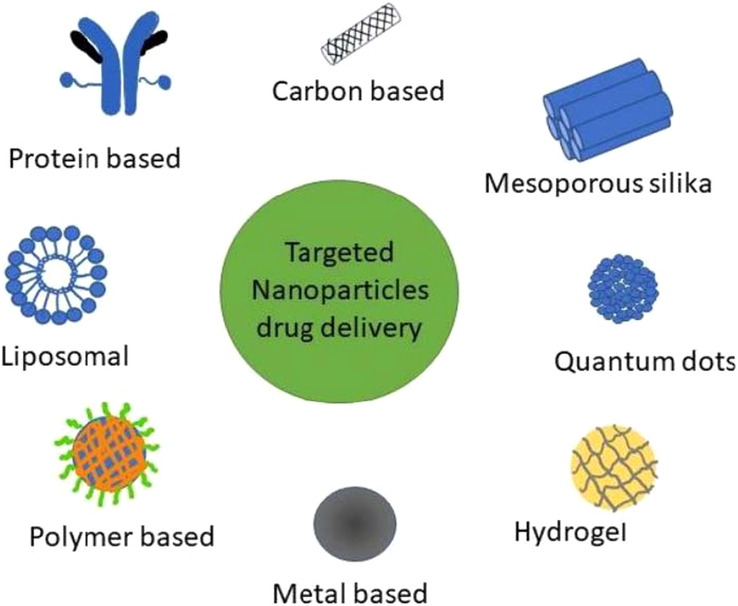
Classification of nanoparticle formulations used in breast cancer research for targeted drug delivery systems (reproduced with permission from Yedi *et al*.^67^).

Different forms of targeted drug delivery systems nanoparticles are utilized for breast cancer research (Fig. 3). NPs can be classified into different types, including liposomal, mesoporous silica, polymer, carbon, metal, and protein-based NPs^66^.

The efficacy of NPs in drug delivery is contingent upon various factors, including their shape, size, content, and other structural characteristics. NPs possess a complex structure consisting of several components, which are designed to achieve therapeutic and/or therapeutic objectives^68^.

Nurse practitioners use many ways to exert an effect on cancer cells:

1. Pharmaceuticals that are shielded against hepatic inactivation, enzymatic decomposition, and fast removal^69^.
2. The functionalization of ligands over-expressed in tumor cells, which are identifiable by receptors (targets), has improved the internalization of cancer cells^68^.
3. It exerts an influence on the progression of cancer by regulating the tumor microenvironment (TME)^70^. Polymers have the ability to detect and react to external stimuli such as light, temperature, ultrasound, and electrochemical triggers, as well as the specific conditions within a tumor, such as pH, enzyme activity, and redox characteristics. This enables them to release drugs in order to overcome these obstacles^70^.
4. Avoided the mechanisms of multiple drug resistance (MDR) efflux transporters^68^.
5. Diminished the occurrence and severity of adverse reactions^71^.
6. The contrast moieties were encapsulated in a carrier, enabling direct viewing of the carrier *in vivo* ^72^. Table 7 showcases a variety of nanoparticles in different forms that have been clinically studied for treating various cancer types^73^.

**Table 7:** Some of the nanomedicines, the subject of clinical studies in recent years, as compiled from clinicaltrials.gov

Nanodrug	Conventional drug	Type of cancer	Clinical Trials.gov Identifier
ONPATTRO	Patisiran	Transthyretin amyloidosis	NCT03862807
AGuIX	Polysiloxane gadolinium-chelates-based nanoparticles	Brain metastases	NCT02820454
VYXEOS	Cytarabine, daunorubicin	Acute myeloid leukemia	NCT04920500
Paclitaxel Nab	Mifepristone	Recurrent breast cancer, Male breast cancer	NCT01493310
	Advanced or metastatic	Azacitidine (Vidaza) Breast cancer	NCT00748553
	Bevacizumab, Gemcitabine hydrochloride	Breast cancer	NCT00623233

## Targeting mechanisms

**Figure 4:**
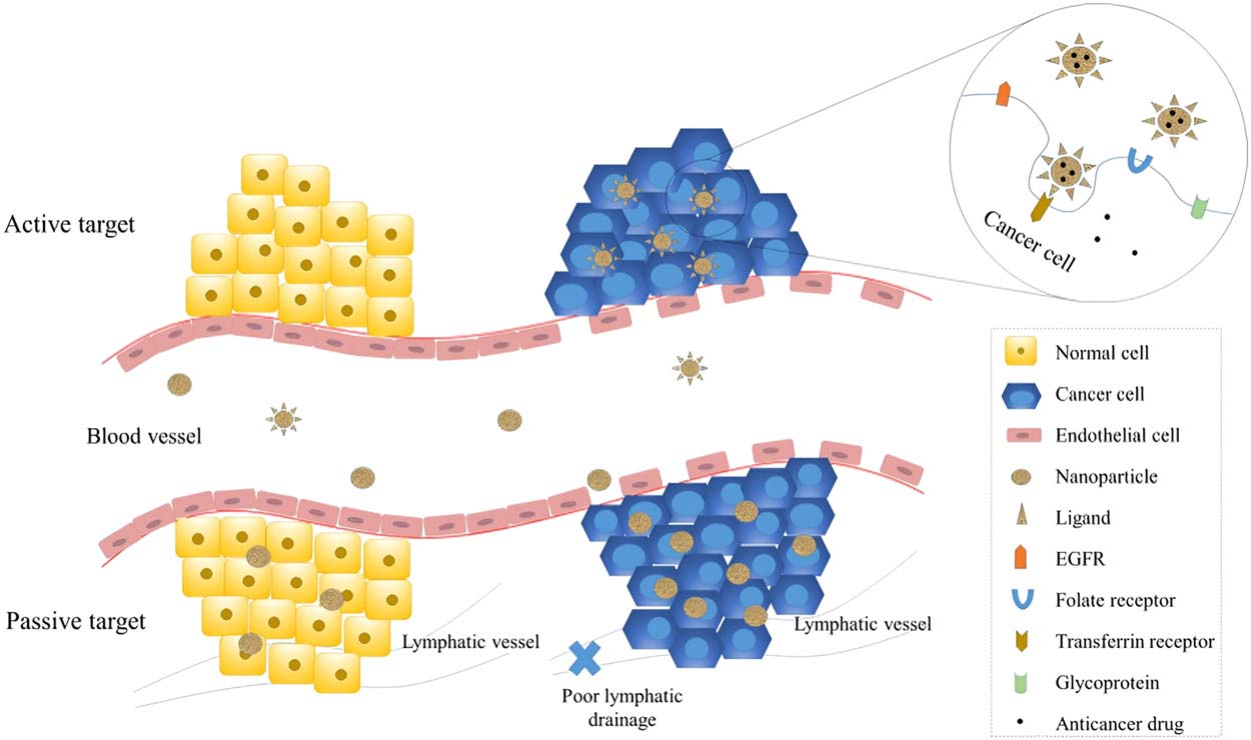
Inactive and active delivery of nanoparticles (NPs) to cancer cells. The targeting of nanoparticles improves the effectiveness of therapy and decreases the toxicity that affects the entire system. The primary method for passively targeting nanoparticles is via the increased Permeability as well as retention phenomenon. This effect takes advantage of the heightened arterial permeability and reduced lymphatic outflow in cancer cells, allowing NPs to target cancer cells selectively without active intervention. Active targeting is accomplished through the interaction between receptors and ligands. The cancer cells possess many types of receptors, including transferrin receptors, folate receptors, glycoprotein receptors (such as lectin), and receptors for the epidermal growth stimulant (reproduced with permission from Yao *et al*.^74^).

Accurate localization is a key feature of small carriers of tumor cells. Employed in medication transportation since it increases the effectiveness of therapy. At the same time, it safeguards regular cells from harmful effects. Several research studies have been conducted to investigate the design of targeting medications based on nanoparticles. To effectively tackle the difficulties associated with cancer cell targeting and the design of nanocarrier systems, having a thorough comprehension of the biology of malignancies and how they interact between cancerous cells and tiny particles is crucial. The targeting techniques can be categorized into two main groups: both inactive and active targeting (Fig. 4).

### Passive targeting

Passive targeting pertains to the power of nanoparticles ranging from 10 to 150 nm to selectively move from the circulatory system into tumor tissue. Nanoparticles have the ability to concentrate on tumor tissue precisely because of the improper formation of binding connections among endothelial cells in newly formed blood arteries within tumors^3^. The process by which nanoparticles passively enter the microenvironment of the tumor is known as the increased permeability and retentivity phenomenon. This phenomenon was first observed around thirty years earlier during the investigation of macromolecule transportation within tumor tissues^3^.

QDs are known for their light-resistant properties, adjustable emission, and high energy conversion efficiency, making them ideal for applications like tumor tissue visualization due to their passive accumulation and improved permeability and retention impact. Hong *et al.* ^75^. utilized a novel near-infrared II (NIR-II) fluorophore, known as six-armed PEG-Ag2S QDs, to visualize subcutaneous xenograft 4T1 mouse tumors. The researchers continuously tracked the distribution of the NIR-II signal in mice over a prolonged time period of time (up to 24 h after injection). The fluorescence emitted by 6PEG-Ag2S QDs in tumors remained high but dropped in other parts of the body and skin within 30 minutes. Until 24 h post-infection, the pharmacokinetics of 6PEG-Ag2S QDs in living organisms showed a remarkable build-up in tumors, with a concentration above 10% ID/gram. This accumulation is attributed to augmented Permeability and retention. The use of near-infrared II quantum dots provides accurate visualization of internal organs, enhanced contrast for tumor imaging, and quicker tumor identification.

Researchers have utilized AuNPs for in-vivo tumor imaging by means of passive targeting. In their study, Lai *et al.* ^76^. demonstrated that gold nanoparticles coated with mercaptoundecanoic acid were able to accurately detect and monitor primary glioma cells in the areas where they were injected into the brains of mice. Moreover, these particles accurately identified the microvasculature associated with tumors. Chitosan nanoparticles have been utilized in certain instances for in-vivo imaging by exploiting the enhanced permeability and retention effect. Nam *et al.* ^77^. described a targeted nanoparticle for tumors that may be used as a versatile imaging scan for optical/MR (MR: magnetic resonance) dual imaging. This nanoparticle is built on Glycol chitosan self-assembles. By employing conjugation and alteration of chemicals conjugation and alteration of techniques, researchers successfully created chitosan nanoparticles that are both stable and tagged with Cy5.5. Researchers have developed a new method to target tumors in living organisms using glycol chitosan nanocrystals. The nanoparticles, encapsulated by Gd(III), have a spherical morphology and are around 350 nm in diameter. They have been found to be efficient in uptake and dispersion within the cytoplasm. After intravenous treatment in animals with tumors, the nanoparticles accumulate abundantly in the tumor, as confirmed by non-invasive fluorescence imaging and magnetic resonance imaging. EPR effect could significantly influence their potential for cancer identification and control.

The dimensions and morphology of nanoparticles significantly impact their EPR impact, making it crucial to consider these characteristics when designing nanoparticle probes for tumor formation. Nanoparticles smaller than ten nm can be cleared by the kidneys, reducing their accumulation in malignant tissue. Anisotropic molecules increase circulation duration due to the reduced likelihood of permeating liver endothelial gaps. A study using silica-coated QDs with different thicknesses investigated the impact of nanoparticle dimensions on particle accumulation in tumor tissue. 12-nm QDs entered tumor tissue without obstruction, while 60-nm QDs leaked out of blood vessels but remained within 10-micrometer vessels^78^.

Upon coming into touch with a biological fluid, nanoparticles will acquire a layer of biological components on their surface, known as a “corona”^79^. The presence of serum proteins on the nanoparticle’s surface (opsonization) significantly alters the way nanoparticles are transported, taken up, and eliminated in the body. Applying PEG as a coating on the surface of a nanoparticle decreases the occurrence of unspecific binding of serum proteins and limits the creation of a protein corona, consequently expanding the duration of nanoparticle circulation. PEGylation of different nanoparticles, like AuNPs and QDs, leads to an extended duration of presence in the bloodstream, as well as gradual build-up in the liver and spleen.

Nanotechnology-based imaging is anticipated to enhance the precision and selectivity of cancer detection while simultaneously decreasing toxicity. In a recent study, Garrigue *et al.* ^80^. demonstrated the utilization of nanoparticles and the EPR effect to create a novel nanosystem for positron emission tomography (PET) imaging. The method utilizes a self-assembling amphiphilic dendrimer that maintains different PET reporting units at its ends. The dendrimer exhibited the ability to autonomously create small, homogeneous nano micelles that are selectively concentrated in tumors, so enabling efficient PET imaging. Thanks to the dendrimeric multivalence and tumor targeting inactive facilitated by EPR, the nanosystem demonstrated enhanced imaging sensitivity and heightened particularity. The ratios of PET signals rose by around 14 times compared with the clinical gold standard, 2-fluorodeoxyglucose. Furthermore, the dendrimer exhibited exceptional safety characteristics and favorable pharmacokinetics for PET imaging. The authors assert that their study makes a valuable contribution to the advancement of dendrimer nanosystems for efficient and auspicious cancer imaging.

### Targeting with intention

Researchers have explored using cell receptor membrane recognition to target tumor tissues and nanoparticle accumulation for tumor imaging. These techniques increase the amount of nanoparticles transported to tumor tissue, enhancing sensitivity in in-vivo tumor detection. Active tumor targeting is more effective than EPR effect-based passive targeting for early cancer detection using high-contrast imaging^81^.

Levenson and Nie’s study demonstrates the use of antibody-conjugated QDs to target prostate-specific membrane antigen (PSMA) for tumor targeting. They analyzed in-vivo imaging outcomes of three QD surface modifications: carboxylic acid (COOH), polyethylene glycol (PEG), and a combination of PEG and prostate-specific membrane antigen-antibody. The results showed that active targeting using a tumor-specific ligand is more efficient and faster than passive targeting in terms of incorporation, retention, and tumor penetration^81^.

A study has shown that regular administration of peptides can effectively target malignant areas in living organisms. The RGD peptide, which binds to the αvβ3 protein, plays a role in cancer angiogenesis and metastasis. A system using gold nanoparticles was developed to target specific cells precisely. The technique involves combining a tumor-homing penetration peptide called iRGD with a legumain-responsive aggregable gold nanoparticle. The compounds, administered intravenously, are specifically attached to the tumor’s blood vessels and distributed throughout the surrounding tissue. iRGD targets tumors through a three-step process: binding to αv integrins on the tumor endothelium, creating a binding motif for neuropilin-1, and controlling cell penetration. This addition enhances the sensitivity of tumor imaging agents and antitumor medicine efficacy^82^.

A study has developed a platform using DNA to create near-infrared sensitive nanoparticles for cancer treatment. The platform consists of complementary DNA strands, 50 × 10 nm gold nanorods, and a PEG layer. The DNA strands, composed of cytosine-guanine (CG) base pairs, provide binding sites for the chemotherapy medication Dox. The drug loading can be adjusted by changing the amount of CG base pairs. The capture strand transports the medication, while the targeting strand targets cells. Gold nanorods are efficient transducers of near-infrared light, making them useful in cancer thermotherapy and denaturing double-stranded DNA when exposed to NIR radiation. This process releases loaded medications at the desired location for chemotherapy^83^.

## Clinical use of nanomaterials in the treatment of cancer

Nanomaterials have shown promising results in therapeutic scenarios, particularly in the delivery of anti-cancer medications. One example is CRLX101, a medication that combines camptothecin with nanoparticles. It was tested in clinical trials for advanced rectal patients with cancer, showing well-tolerance and a complete pathologic response^84^. A phase I-IIa clinical trial investigated the effectiveness of CRLX101 in combination with bevacizumab in metastatic renal cell patients with cancer. A phase 1/2a trial tested the effectiveness of CRLX101, a nanopharmaceutical combining a cyclodextrin-containing polymer and camptothecin, on patients with advanced solid tumor malignancies^85^.

### Types of nanoparticles used for each organ cancer

#### Breast cancer

*Polymeric nanoparticles*: Used for targeted drug delivery, often carrying chemotherapeutic agents like doxorubicin. The surface of these nanoparticles can be modified with antibodies targeting receptors over-expressed in breast cancer cells, such as HER2.

*Liposomes*: Liposomal formulations (e.g. liposomal doxorubicin) are used to reduce toxicity and improve the efficacy of chemotherapy.

#### Lung cancer

*Carbon nanotubes*: Engineered for targeted delivery of chemotherapeutic agents directly into lung cancer cells, potentially through inhalation therapies that target the lung specifically.

*Gold nanoparticles*: Utilized in photothermal therapy, where they are targeted to lung cancer cells and then heated with infrared light to kill cancer cells without harming surrounding healthy tissue.

#### Prostate cancer

*Gold nanoparticles*: Can be functionalized with prostate-specific membrane antigen (PSMA) ligands to target and image prostate cancer cells specifically.

*Magnetic nanoparticles*: Used in hyperthermia treatment and as MRI contrast agents, targeted to prostate cancer cells with specific antibodies.

#### Brain cancer (glioblastoma)

*Magnetic nanoparticle*s: Functionalized to cross the blood-brain barrier (BBB) and target glioblastoma cells, used in conjunction with MRI for diagnosis or hyperthermia for treatment.

*Polymeric nanoparticles*: Designed to bypass the BBB and deliver chemotherapeutic drugs directly to brain tumors, often modified with targeting moieties that recognize brain tumor cells.

#### Pancreatic cancer

*Polymeric-based nanoparticles*: These nanoparticles can be designed to release their drug load in response to the specific microenvironment of pancreatic tumors, such as low pH or high levels of certain enzymes.

*Metallic nanoparticles*: Especially gold nanoparticles have been explored for the photothermal treatment of pancreatic cancer, where they are targeted to the tumor and then irradiated to produce heat and kill cancer cells.

## Future prospects and current challenges

Magnetic nanoparticles, with their nanoscale dimensions, heightened reactivity, and enhanced surface area, can potentially cause harmful effects on human cells or tissues. They can enter the human body through ingestion or inhalation and migrate to different organs and tissues. The environmental impact of nanoparticles is recognized, and efforts are being made to develop harmless materials. Nanoparticles, like dendrimers, can disrupt enzymatic assays or generate excessive charges, potentially affecting cell membrane integrity or cell death. Despite their benefits in cancer therapeutics and imaging, concerns remain about their potential toxicity and adverse effects^86^. Nanoparticles can accumulate in organs like the liver and kidneys, potentially causing health issues. However, their exact impact on the human body is not yet fully understood. Experts are concerned about the toxicity and potential hazards they pose to the environment and human health, particularly metallic nanoparticles, which can interact with other pollutants and enhance their hazardous effects. The exact impact of these accumulations on the human body is not yet fully understood. Nanomedicine has unique adverse impacts compared to conventional medications due to its varying toxicity and ability to traverse multiple body barriers, including the blood-brain barrier. Nanoparticles, which are extremely small, can also pass through cell membranes, intracellular organelles, and even the nucleus, affecting various organs and potentially causing various effects on the brain^87^. Nanomedicine balances toxicity and efficacy in therapeutic treatments by combining biological, chemical, and physical characteristics. It evaluates nano-compound biodistribution in pre-clinical and clinical investigations, considering the unique characteristics and limitations of each Technology. It helps assess immediate build-up in cells, organs, and tissues, ensuring a harmonious balance between toxicity and efficacy^88^.

Green nanotechnology is a promising alternative to harsh methods and toxic chemicals used in nanoparticle synthesis. It involves integrating microbes and plants, resulting in highly optimized, low-toxicity, and environmentally friendly nanoparticles. These nanoparticles are safe for humans and the ecosystem. Intelligent Ag nanoparticles have unique scattering characteristics, promoting resistance against bacteria and aggregation. Their enhanced dispersion features offer potential for smart nanotechnologies in various domains. This approach eliminates the need for foreign stabilizing or coating agents^89^. Nanoparticles can cross physiological barriers, like blood-brain, and interact with cell membranes, organelles, and nuclei, influencing drug administration in nanomedicine. However, toxicity is largely influenced by the composition of the nanoparticle formula^90^.

The biodistribution of nanomedicine is crucial in pre-clinical and clinical investigations. Nanoparticles have been found to accumulate on the skin, demonstrating resistance against bacteria and promoting aggregation. This is due to their surface ligands resembling the bacterial membrane, leading to a notable antibacterial impact. The enhanced dispersion features of this invention offer excellent prospects for the utilization of smart nanotechnologies across diverse domains. Research has shown that each Technology has unique characteristics and limitations in real-time substance accumulation^91^. Nanoparticles of heavy minerals, such as tin, mercury, and lead, are recognized for their stability and resistance to degradation, resulting in significant environmental toxicity. Research has evaluated the overall toxicity, ability to cause genetic damage, and potential to cause cancer in rats exposed to both tannic acid and iron (III) nanoparticles, as well as in both normal and malignant human cells^92^. The future of nano-based materials for cancer therapeutics lies in the development of biocompatible nanomaterials with reduced toxicity and improved biodistribution.

## Conclusion

The development of a more potent cancer therapy is the primary driving force behind the establishment of cancer nanotechnology. Many restrictions exist with the most widely used cancer treatment procedures, such as radiation therapy and chemotherapy. The restrictions have been partially removed with the advent of nanotechnology. Research on nanoparticles, liposomes, dendrimers, and nanoshells is still ongoing in the field of nanotechnology. An expanding toolkit for treating cancer is cancer nanotechnology. Nanotechnology offers many important advantages in cancer diagnosis compared to traditional methods, including:

1. Early detection: Nanotechnologies allow early detection of cancer at the level of cells or macromolecules, allowing early intervention and thus increasing the chances of recovery.
2. Accuracy and specificity: Nanotechnology can improve the accuracy and specificity of diagnosis, as nanomaterials can be used to specifically target cancer cells without affecting surrounding healthy tissue.
3. High sensitivity: Nanotechnologies are more sensitive in detecting subtle changes in the body associated with cancer, which enables it to be detected at early stages and increases the effectiveness of treatment.
4. Nano-X-ray imaging: Nano-X-ray imaging allows the viewing of tiny structures inside the body with greater accuracy than traditional imaging, which helps in better diagnosing and staging cancer.
5. Precise guidance and delivery of treatment: Nanotechnology can be used to precisely direct and deliver treatment to areas affected by cancer without affecting healthy tissue, reducing side effects.

Future directions for nanotechnology innovation in cancer therapy include the creation of self-organizing and reactive systems. In addition to continuing to be of assistance, medical science will continue to be greatly benefited by nanotechnology in the years to come. This will be a very exciting period for nanomedicine.

## Ethical approval

Ethics approval was not required for this review article.

## Consent

Informed consent was not required for this review article.

## Source of funding

Not applicable.

## Author contribution

O.O.L.: conceptualization, data curation, writing—original draft preparation, writing—reviewing and editing. I.B.A.: writing—reviewing and editing, visualization, supervision. H.H.: writing—reviewing and editing, visualization, supervision. W.H.: writing—reviewing and editing, visualization, supervision. S.Z.: writing—reviewing and editing, visualization, supervision. A.B.A.: writing—reviewing and editing, visualization, supervision. M.B.: writing—reviewing and editing, visualization, supervision. A.A.A.: data curation, writing—original draft preparation, writing—reviewing and editing.

## Conflicts of interest disclosure

The authors declare no conflicts of interest.

## Research registration unique identifying number (UIN)

Not applicable.

## Guarantor

Ali Alnazza Alhamad.

## Data availability statement

Not applicable.

## Provenance and peer review

Not applicable.
